# Detection of Partial AZFc Microdeletions in Azoospermic Infertile Men Is Not Informative of MicroTESE Outcome

**DOI:** 10.22074/ijfs.2019.5397

**Published:** 2018-10-02

**Authors:** Azam Miraghazadeh, Mohammad Ali Sadighi Gilani, Fakhredin Reihani-Sabet, Azadeh Ghaheri, Parnaz Borjian Boroujeni, Mohammadreza Zamanian

**Affiliations:** 1 Department of Molecular and Cellular Sciences, Faculty of Advanced Sciences and Technology, Science and Research Branch, Islamic Azad University, Tehran, Iran; 2 Department of Genetics, Reproductive Biomedicine Research Center, Royan Institute for Reproductive Biomedicine, ACECR, Tehran, Iran; 3 Department of Andrology, Reproductive Biomedicine Research Center, Royan Institute for Reproductive Biomedicine, ACECR, Tehran, Iran; 4 Department of Epidemiology and Reproductive Health, Reproductive Epidemiology Research Center, Royan Institute for Reproductive Biomedicine, ACECR, Tehran, Iran

**Keywords:** Azoospermia, Infertility, Microsurgery, Sperm Retrieval, Y Chromosome

## Abstract

**Background:**

Microdeletions of the Yq chromosome are among the most frequent genetic etiological factor of male infertility which spans the azoospermia factor regions (AZFa, AZFb and AZFc). Microdeletions are mostly seen in the AZFc region and usually cover genes actively involved in spermatogenesis. Partial AZFc microdeletions may also occur with various spans, namely gr/gr, b2/b3 and b1/b3. It is known that the outcome of microtesticular sperm extraction (TESE), the surgical process for sperm retrieval from the testis in infertile azoospermic men, may be pre- dicted based on the type of AZF microdeletion. We therefore aimed to evaluate the correlation between partial AZFc microdeletions and microTESE results.

**Materials and Methods:**

In this cross-sectional study, 200 infertile azoospermic men referred to the Royan Institute were examined for the presence of partial AZFc microdeletions before undergoing microTESE. Partial AZFc micro- deletions were detected by multiplex polymerase chain reaction (PCR) of seven different sequence-tagged site (STS) markers. The data were analyzed with the Chi-square test.

**Results:**

Among the 90 patients (45%) with a positive microTESE outcome, 9 (10%) showed a partial microdeletion in AZFc region. Of the 110 (55%) patients with a negative microTESE outcome, 7 (6.3%) had an AZFc partial micro- deletion. With respect to the span of the microdeletions, among the 200 patients, 11 (5.5%) were gr/gr and 5 (2.5%) were b2/b3. Statistical analysis showed no significant difference between the patients with and without partial AZFc microdeletions with respect to microTESE outcome.

**Conclusion:**

Partial AZFc microdeletions is not a predictor of microTESE outcome in azoospermic men.

## Introduction

Infertility, defined as the failure to conceive after one year of regular intercourse, is caused by male factors in 15% of cases (1, 2). Genetic factors play a role in at least 10% of male infertility cases, of which chromosomal disorders and Y chromosome microdeletions are the most prevalent (3). The azoospermia factor (AZF) region is located on the long arm of Yq and contains important genes involved in the process of spermatogenesis. Microdeletions in the AZF regions (a, b and c) usually lead to azoospermic/ severe oligospermic male infertility (4).

AZFc is the most common Yq microdeletion (5) spanning about 3.5 Mbp of Yq and is divided into three smaller subcategories of partial microdeltions, namely gr/gr, b2/ b3 and b1/b3 (6, 7). In the classic AZFc microdeletion (b2/b4), four copies of DAZ are removed leading to spermatogenic failure (5). The gr/gr deletion, the most frequent partial AZFc deletion, removes around half of the b2/b4 region and may be clinically presented by various phenotypes based on ethnic and geographic origin (8-10). The b2/b3 and b1/b3 partial deletions are rare and have been studied less often (11, 12).

The chance of sperm retrieval during surgical or microsurgical procedures such as testicular sperm extraction (TESE), known as microTESE, may be predicted if there is a microdeltion in the AZF region (13). This chance is virtually zero in cases with AZFa and AZFb, however, in AZFc microdeltion carriers, this may increase to more than 50% (14). Since there was no previous report on the chance of sperm retrieval in men with AZFc partial microdeletions, we first determined the frequency of such deletions in a cohort of azoospermic/severe oligospermic men and then assessed its relationship with microTESE outcome.

## Materials and Methods

This cross-sectional study was conducted during 2013-2014. A total of 200 infertile men with azoospermia/severe oligospermia, as candidates for microTESE surgery at the Royan Infertility Center, were included. The study was approved by the Ethics Committee of the Royan Institute (IR.ACECR.ROYAN.REC.1395.1) and written consent was obtained from the patients. The patients were checked for AZF full microdeletions and reported as normal based on the EAA/EMQN best practice guidelines for molecular diagnosis of Y-chromosomal microdeletions (13). Infertile patients with obstructive azoospermia, varicocele, cryptorchidism, endocrine problems, history of chemotherapy or radiotherapy and abnormal karyotype were excluded from the study. MicroTESE candidates were then checked for the presence of AZFc partial microdeletions.

PAXgene Blood DNA kit (Qiagene, Germany) and the salting out method were used for DNA extraction. Quality and concentration of extracted DNA from peripheral blood was checked by Nanodrop Spectrophotometer 2000 (Thermo Scientific, USA). AZFc partial deletions were analyzed using multiplex polymerase chain reaction (PCR) of seven sequence-tagged site (STS) markers as previously described (7). In brief, STS markers for each AZFc subregion (gr/gr, b2/b3 and b1/b3) were selected and specific primers with predetermined products size were designed (Table 1). Two multiplex PCR reactions (A and B) were prepared by mixing 0.5 μl of 10 mM dNTP mix (Bioron Germany), 0.3 μl of Taq DNA polymerase (Bioron, Germany), 0.55 μl of 15 mM MgCl_2_ and 0.25 μl of 10 pM forward and reverse primers in a total volume of 25 μl. For mix A, the PCR cycling conditions were 94˚C for 4 minutes, 40 cycles of 94˚C for 30 seconds, 64˚C for 40 seconds and 72˚C for 45 seconds and a final extension at 72˚C for 10 minutes. For mix B, the annealing temperature was set at 41˚C. Mix A contained primers for the 1191, 1291 and 1258 STS markers while mix B contained primers for STS markers 1161, 1197, 1206 and 1201. DNA from healthy male and female controls were used as internal positive and negative controls. The PCR products were separated on 3% agarose gels stained with Sybr green (ABM, Germany).

Statistical analyses were performed using SPSS 18.0 statistical software (SPSS, Inc., Chicago, IL, USA). Continuous variables were analyzed using the independent sample t test and categorical variables were analyzed using chi-square test. P<0.05 was considered statistically significant.

**Table 1:** The sequence-tagged site (STS) markers included in each multiplex polymerase chain reaction (PCR) mix. Each marker and its relationship to azoospermia factor c (AZFc) subregions, its related primer set and product size are shown


	Marker	b2/b4	b2/b3	gr/gr	b1/b3	Primer sequence (5ˊ-3ˊ)	Product size (bp)

Mix A	SY1197	+	+	+	_	F: TCATTTGTGTCCTTCTCTTGGA	435
						R: CTAAGCCAGGAACTTGCCAC	
	SY1161	+	+	+	_	F: CGACACTTTTGGGAAGTTTCA	330
						R: TTGTGTCCAGTGGTGGCTTA	
	SY1201	+	+	+	+	F: CCGACTTCCACAATGGCT	677
						R: GGGAGAAAAGTTCTGCAACG	
	SY1206	_	+	+	+	F: ATTGATCTCCTTGGTTCCCC	394
						R: GACATGTGTGGCCAATTTGA	

Mix B	SY1191	_	_	+	_	F: CCAGACGTTCTACCCTTTCG	385
						R: GAGCCGAGATCCAGTTACCA	
	SY1291	_	+	_	_	F: TAAAAGGCAGAACTGCCAGG	527
						R: GGGAGAAAAGTTCTGCAACG	
	SY1258	+	+	+	+	F: AACCCCATCTCTAGCAAAAATATG	930
						R: TAGGTGACAGGGCAGGATTC	


**Table 2:** The clinical characteristics of infertile men


Patients groups	Age(Y)	FSH(mIU/mL)	LH(mIU/mL)	Testosterone(mg/mL)

TESE positive n=90	39.19 ± 6.6	22.87 ± 17.05	11.4 ± 8.20	3.86 ± 4.95
TESE negative n=110	39.22 ± 6.22	24.71 ± 15.61	15.12 ± 10.66	3.59 ± 1.97


Values are mean ± SEM (P=0.974, P=0.431, P=0.006, P=0.627, respectively). FSH; Follicle-stimulating hormone, LH; Luteinizing hormone, TESE; Testicular sperm extraction. Normal ranges: FSH: 2-10 mIU/mL, LH: 1.0-9.5 mIU/mL, Testosterone: >2.4 mg/mL.

## Results

**Fig.1:**
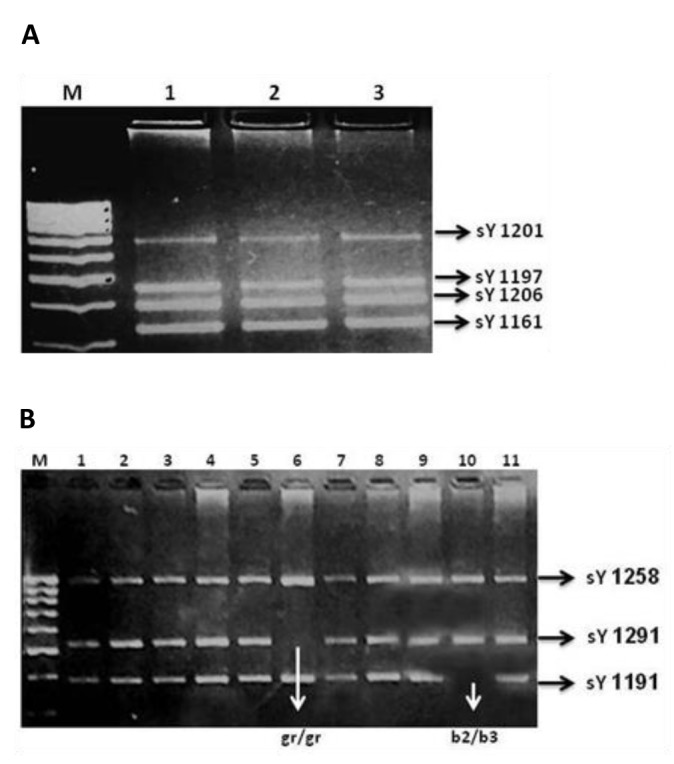
Results for multiplex polymerase chain reaction (PCR). A. Mix A, the band sizes match the relevant sequence-tagged site (STS) markers. No deletion was detected in three samples and B. Mix B, sample 6 is showing a missing band for sY1291 which is representative for a gr/gr deletion while sample 10 has a missing band for sY1191 which is indicative of a b2/b3 deletion in the azoospermia factor c (AZFc) region.

**Fig.2:**
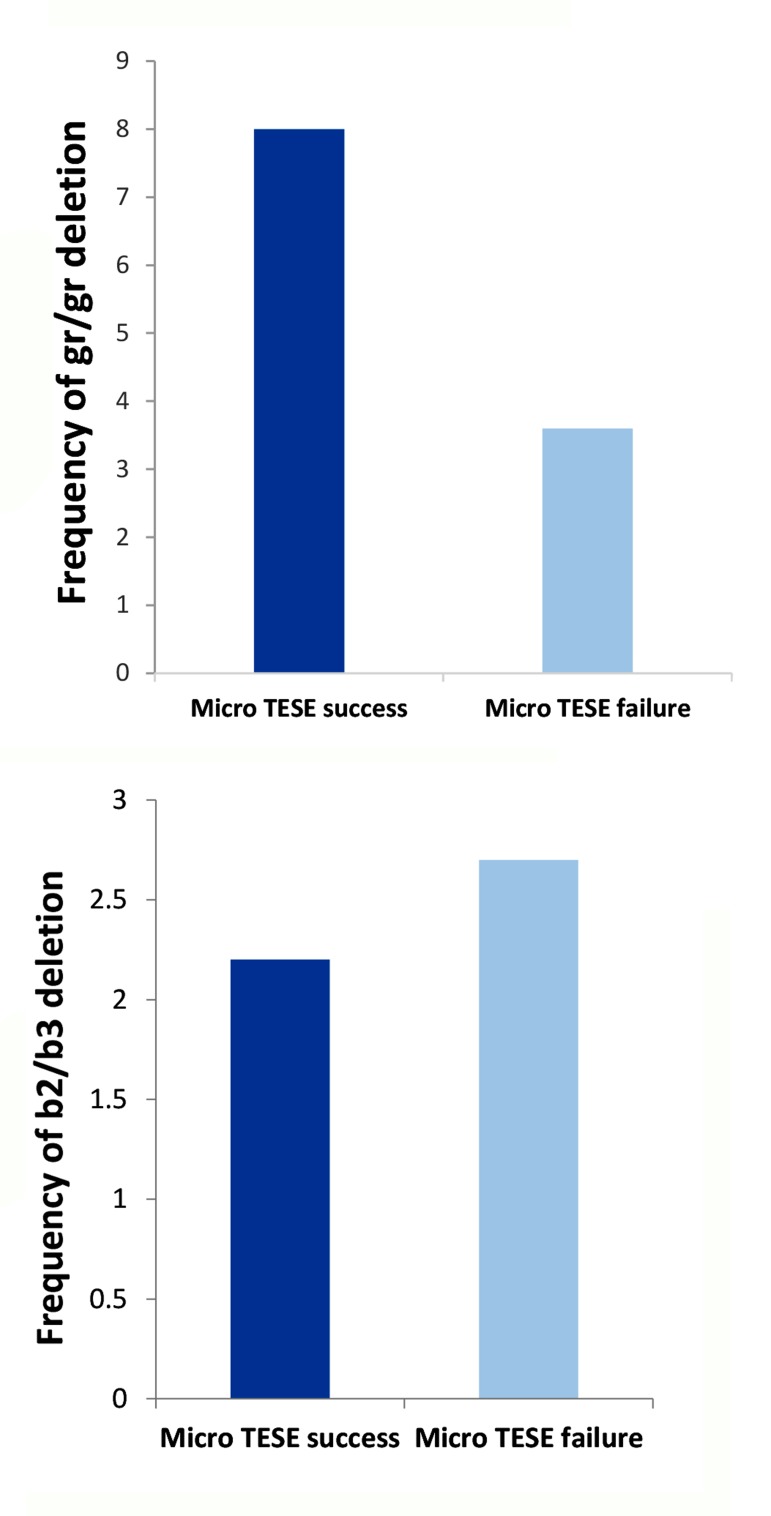
Partial azoospermia factor c (AZFc) microdeletions in infertile men with micro testicular sperm extraction (TESE) surgery. A. Percentage of gr/gr deletions. Comparison showed no significant difference (P=0.201) in those with successful micro TESE compared to those with failed surgery and B. Percentage of b2/b3 microdeletion. Also no significant difference was detected between those with and without sperm retrieval during micro TESE surgery (P=0.82).

Demographic characteristics of the patients including age and hormonal profile are summarized in Table 2. Of the 200 infertile men, 90 cases had a successful microTESE. In assessing partial AZFc deletions, we identified 11 cases of gr/gr and 5 cases of b2/b3 microdeletions among all cases, however, no b1/b3 partial AZFc microdeletion was identified. In samples with a gr/gr deletion, a 527 bp PCR product representing the sY1291 STS marker was missing while those carrying b2/b3 deletions lacked a 385 bp product for the sY1191 STS marker (Fig .1). We detected partial AZFc deletions in 9 and 7 cases among successful and failed microTESE groups respectively (Table 2). There was no statistically significant difference in the frequency of AZFc partial deletions between successful and failed microTESE groups (Fig .2). For gr/gr deletion the frequencies were 8% and 3.6% for successful and failed groups respectively. Finally, for b2/b3 deletion, we detected a frequency of 2.2% in successful group for sperm retrieval versus 2.7% in failed group (Table 3).

**Table 3:** AZFc partial deletions in infertile men based on microTESE outcome. The number and percentage of AZFc partial deletions in each category are shown


	AZFc partial deletion		
Micro TESE result	n	gr/gr	b2/b3	Total

Successful	90	7 (8%)	2 (2.2%)	9 (10%)
Failed	110	4 (3.6%)	3 (2.7%)	7 (6.4%)
P value	-	0.201	0.820	0.346
Total	200	11 (5.5%)	5 (2.5%)	16 (8%)


AZF; Azoospermia factor and TESE; Testicular sperm extraction.

## Discussion

Infertile men with azoospermia/severe oligospermia are usually selected for sperm retrieval surgeries such as microTESE for further assisted reproductive technology (ART) procedures. It is usually recommended to determine AZF microdeletions before surgery to predict the chance of microTESE success (13). For cases carrying AZFa and AZFb microdeletions, this chance is considered as virtually zero while for those with AZFc deletions it can reached up to 50% (15). In AZFc cases, there is also a chance for transmission of infertility to the male offspring (2).

Previous reports on the importance of AZFc partial deletions such as gr/gr and b2/b3 are controversial and seemingly due to ethnic heterogeneity and differences in genetic backgrounds (7-12). Ferlin et al. (7) reported the gr/gr deletion as a risk factor in infertile men of a Caucasian population. In a study on the Sri Lankan population, the gr/gr deletion frequency was reported as equal (4.2%) in both fertile and infertile groups and therefore found not to be associated with spermatogenesis (16). On the contrary, Eloualid et al. (11) and choi et al. (17) reported contributory effects on spermatogenesis for b2/b3 and gr/gr deletions in other populations.

Giachini et al. (18, 19) examined infertile Italian men and detected a higher frequency for the gr/gr deletion in the oligo/azoospermic group but found no difference for the b2/b3 deletion. Furthermore, in a previous study on 100 Iranian infertile men, the gr/gr deletion was linked to spermatogenic failure (20). In contrast, Stahl et al. (10) emphasized that the gr/gr deletion is not an appropriate factor for predicting impaired spermatogenesis. Therefore, effects of partial AZFc microdeletions on spermatogenesis as well as the chance for sperm retrieval during microTESE has remained inconclusive. Given that most of these studies had relatively small sample sizes, we examined this association on a relatively larger sample set of 200 patients. The frequency of AZFc partial deletions for patients with successful and failed microTESE outcome were 10% and 6.4% respectively, further questioning the association, similar to some previous studies (10, 16, 21) who reported a lack of association between AZFc partial deletions and spermatogenic failure. No significant difference was also observed when gr/gr and b2/b3 deletions were considered separately. In present study, one major limitation is the relatively small sample size which could limit the statistical power and be the reason for the absence of significant relationship between micro TESE results and AZFc microdeletions. In addition to a bigger sample size, it is recommended to consider gene content in partial AZFc deletions in future studies.

## Conclusion

We found no evidence for partial AZFc microdeletion influencing outcome of sperm extraction during microTESE. A larger association study may reveal the diagnostic value of such deletions in men who are candidates for microTESE.
